# Exploring GPs’ prioritisation dilemmas — ‘quick wins’ versus ‘eating the frog’: a qualitative study in primary care

**DOI:** 10.3399/BJGP.2025.0628

**Published:** 2026-07-28

**Authors:** Andrew McClarey, Agata Dunsmore, Duncan McNab, Samantha Eve Smith

**Affiliations:** 1 Scottish Centre for Simulation and Clinical Human Factors, NHS Forth Valley, Larbert, UK; 2 School of Medicine, University of Edinburgh, Edinburgh, UK; 3 Frew Terrace Surgery, Irvine, UK; 4 School of Medicine, University of Dundee, Dundee, UK

**Keywords:** decision making, education, general practice, prioritisation, workload

## Abstract

**Background:**

Prioritisation is an increasing part of a GP’s workload, although learning this skill is usually left to on-the-job training. Little is known about how experienced GPs prioritise their workload.

**Aim:**

To explore the factors that experienced GPs consider when prioritising their acute workload.

**Design and setting:**

This qualitative research study used focus groups of GP educators based in central Scotland to explore the factors that they consider when prioritising their acute workload.

**Method:**

Participants were recruited through convenience sampling. The key inclusion criterion was being involved in the supervision of GP registrars. Five focus groups were carried out, and transcripts were analysed using reflexive thematic analysis to identify the dilemmas that GPs face when prioritising their workload.

**Results:**

Thirty-nine participants took part. The following five themes emerged from the data: 1) system awareness and adapting to demand, whereby GPs maintain an awareness of their work system and adapt their consultation style to demand; 2) time management, which is where they make decisions in an order that manages their time efficiently; 3) familiarity with patients, which enables them to make quick decisions about patient care, although this can lead to bias in decision making; 4) relationships, whereby they rely on their therapeutic relationships with patients and working relationships with colleagues when making decisions; 5) personal preferences, which are important such as managing the simplest or the most complex presentations first.

**Conclusion:**

Prioritisation is an increasingly important skill in modern general practice. It is crucial that those involved in prioritisation are given the time and tools necessary to adequately develop this skill.

## How this fits in

This is the first study to look at the factors that experienced GPs consider when prioritising their acute workload. Several themes have emerged that highlight the importance of prioritisation training in general practice. These themes could be used to teach prioritisation decision making to GP registrars or in the creation of continuing professional development resources for experienced GPs.

## Introduction

General practice in the UK remains under significant pressure, with demand continually outstripping supply.^1^ To mitigate this pressure, many surgeries have adopted a system of triage and prioritisation of their workload to best allocate the available resources in an equitable way. The number of surgeries using prioritisation techniques has increased significantly since the COVID-19 pandemic,^2^ meaning that this is an increasingly prevalent part of a GP’s work. Triage can be a labour-intensive process and can place a high degree of cognitive load on the clinician, particularly for those less clinically experienced or those unfamiliar with the surgery.^2,3^ This is especially pertinent given the degree of burnout reported among the GP workforce.^4^ Despite this, and the fact that the General Medical Council (GMC) expects doctors to be able to prioritise and reprioritise their workload,^5^ this important aspect of clinical care is often not formally taught during GP training. Obtaining this skill is left to on-the-job learning, which may create inequity in learning opportunities.

Experienced GPs have adapted to facilitate this new way of working, yet little is known about how they make prioritisation decisions when managing their acute workload. A better understanding of GP prioritisation would allow the development of teaching tools and resources to improve prioritisation skills for GP registrars. This would also allow more established GPs to compare their decision-making processes with those of others to share good practice, increase efficiency, and promote sustainable working practices.

### Aim

This study aimed to explore the factors that experienced GPs consider when prioritising their acute workload.

### Theoretical framework

The Systems Engineering Initiative for Patient Safety 2.0 (SEIPS) framework was used to form the basis of the theoretical framework of this study. The SEIPS framework is a human factors engineering model used to promote patient safety through analysis of healthcare systems.^6^ It views healthcare organisations as complex interacting systems with interactions between people, tasks, tools, and technologies, the physical environment and organisational structure. SEIPS has been used in various aspects of the healthcare system including in interprofessional education and workplace process mapping.^7,8^ It was chosen owing to its emphasis on ‘work as done’ rather than ‘work as imagined’, which makes it particularly useful in exploring GPs’ prioritisation decisions.

## Method

### Study design

This qualitative study was conducted from a constructivist viewpoint to explore the factors that experienced GPs consider when prioritising their workload.

### Participant selection and recruitment

A convenience sampling approach was used to recruit participants from trainer groups who already met regularly. Four training programme directors (TPDs) from the central regions of Scotland were emailed with details of the study to invite their group of GPs, who supervise registrars, to participate. TPDs are responsible for the coordination and delivery of the GP training programme. Participants were eligible to participate if they met the Scotland deanery inclusion criteria to become an educational supervisor as detailed in Box 1. Participants were provided with a participant information sheet before obtaining written consent. Participants could withdraw at any point, and their data withdrawn provided it had not been embedded within the analysis.

**Box 1.** The requirements to become an educational supervisor in general practice

Educational supervisor requirements
A fully qualified GP who:
Has obtained a Member of the Royal College of General Practitioners (MRCGP) qualification
Has been working for a minimum of 2 years’ post-qualification
Works a minimum of four GP sessions per week

### Data collection

Five focus groups were carried out between February 2023 and April 2023. Four of the focus groups were chaired by the first author and the other by the second author.

Participants initially undertook a Tactical Decision Game (TDG) exploring a duty doctor scenario in a GP surgery.^9^ A TDG is a tabletop simulation exercise during which participants make prioritisation decisions based on incomplete information given through short written vignettes. Participants were given a list of clinical scenarios and were asked to rank the presentations in the order they would carry them out, initially as individuals and then as a group. Scenarios included acute presentations such as a 51-year-old male with chest pain as well as medication and medical certificate requests. This allowed the construction of prioritisation dilemmas and gave direction to the focus group discussions.

A structured debriefing of the decisions made was then carried out, which acted as an elicitation technique for further group reflection and discussion. Following the TDG, the first and second authors explored the factors that the participants considered when making prioritisation decisions. Participants could reflect on their decision making during the TDG as well as their own clinical practice. Following each focus group the topic guide was iteratively reviewed to capture new themes to be explored (Supplementary Table S1). The focus groups were audio-recorded, transcribed verbatim, and then de-identified. Focus groups were chosen as they enable interaction between participants and can facilitate discussion among participants with differing opinions, yielding a deeper understanding of triage decision making. Data collection ceased when there was sufficient information power to address the research aim.^9,10^

### Data analysis

Data analysis occurred concurrently with data collection in iterative cycles using reflexive thematic analysis.^11^ Following the steps of reflexive thematic analysis, the first author performed initial familiarisation with the transcripts and generated initial codes. The other three authors each independently coded a single focus group transcript. Any discrepancies were discussed, with the first author making a final decision where consensus could not be reached. The final two transcripts were coded by the first author. The evolving codes and themes were discussed regularly by the research team. Data analysis ceased when the research team were satisfied that the themes adequately captured the data collected. Data analysis was performed using securely shared documents on Microsoft Word.

During data analysis, the constructed themes began to converge around the concept of prioritisation dilemmas. A dilemma is *‘a situation in which a difficult choice has to be made between alternatives’*.^12^ Prioritisation dilemmas were prevalent within the dataset, and this concept therefore became another sensitising lens through which the data were viewed.

### Reflexivity

All of the research team members were practising Scotland-based GPs with a shared interest in medical education, each holding educational, research, and clinical roles. Being practising GPs will have influenced the way that the team approached the topic, interacted with participants, and interpreted the data. The team’s common interest with participants will have facilitated rapport during focus groups and will have allowed the team to gain a deeper understanding of the data. That said, the team remained mindful throughout of how their own experiences and narratives could have influenced the data collection and interpretation. The first author held the view that prioritisation was mainly done on the clinicians’ assessment of the acuity of the presentation. The basis of this was having worked in several roles in secondary care where this method of prioritisation is encouraged. The first author had worked in general practices as a GP, although prioritisation had never been a significant part of these roles, nor had the first author been formally trained on prioritisation.

Reflexivity details for the other team members are included in Supplementary Box S1. The first author kept a reflexive journal throughout the research, and the group had reflexive discussions during the research meetings.

## Results

A total of five focus groups were carried out with 39 participants, from a range of practice demographics in the central regions of Scotland. Participants had an average of 9.6 years (range 6 months to 23 years) of experience as a GP educational supervisor. Individual participant demographics, including gender, years of post-graduation experience, years a GP trainer, and practice mean deprivation score, are detailed in Table 1.

**Table 1.** The demographics of the focus group participants

Characteristic	Description
Self-described gender	
Male	23
Female	16
Years since graduation from medical school	Mean 24.6 years, range 5–38 years
Years as a GP trainer	Mean 9.6 years, range 0.5–23 years
Practice mean deprivation score^a^	
3^rd^ decile	1
4^th^ decile	9
5^th^ decile	11
6^th^ decile	10
7^th^ decile	5
8^th^ decile	3

^a^Practice mean deprivation score is the individual practice weighted mean Scottish Index of Multiple Deprivation (SIMD) decile. This decile was calculated from data provided by Public Health Scotland as at 2022–2023.^26^ SIMD 1 represents the most deprived decile and SIMD 10 represents the least deprived decile.

The following five themes were generated from the data: 1) system awareness and adapting to demand; 2) time management; 3) familiarity with patients; 4) relationships; and 5) personal preferences. Dilemmas were present within each of the five themes (Figure 1).

**Figure 1.**
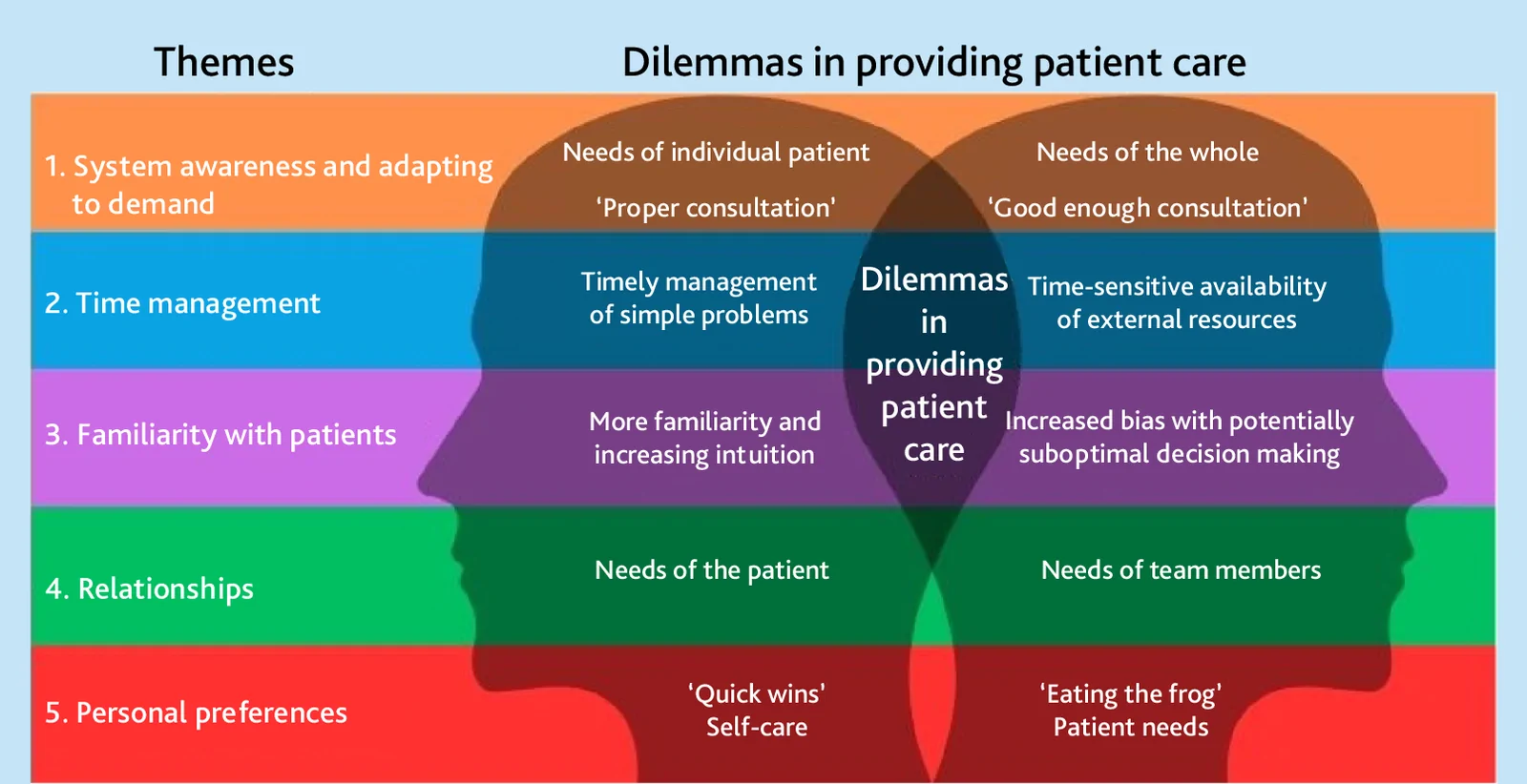
A graphic illustrating the tensions that exist in making prioritisation decisions regarding patient care.

### Theme 1: System awareness and adapting to demand

#### Consultation style

When the system is under increased demand, the participants described adjusting their consultation style to enable them to be more time efficient. Participants adopted a closed consultation style to safely deal with the presenting problem without exploring other matters extraneous to the presenting complaint. They also described being more likely to manage presentations either in whole or partially over the telephone with a view to arranging a further review at a time when there is less demand on the service if required.

Participants reported that increased demand often led to higher prescribing rates as a more expedient response in pressured environments:

*‘… your prescription levels go up for antibiotics, painkillers … when we’re under pressure …’* (P32)

Participants also reflected that their approach is different from how GP registrars might approach working:

*‘So, they* [registrars] *are going to see every encounter as an individual encounter that they need to close off safely. Whereas we’re thinking okay, there’s twenty patients here that we need to sort out, how are we going to sort out those twenty patients?’* (P25)

#### Taking an overall view of work system

Participants discussed that their experience resulted in them reviewing the patient list as a whole, based on the mix and quantity of patient presentations, which determined the approach to the individual patient. One participant articulated the challenges of this theme regarding educating GP registrars:

*‘… you want to show them how it should be done, like a proper consultation. And then there’s also the, what you do on the bad days, and you just get a quick history and go, right, okay, I know you already. Let’s give you the antibiotics and move on … I sort of feel I should teach them the proper way to do it before we start cutting the corners.’* (P35)

The participant above discussed the importance of a structured consultation, particularly for GP registrars. However, participants also described that more experienced GPs consider the system in which this patient is presenting. For example, the ‘quick history’ enables a working diagnosis and ruling out concerning features without going into a structured and comprehensive consultation. In a system with increased demand, this is an example of how participants enabled safe clinical care. Participants describe the challenges that registrars face when moving from managing a single patient in a *‘proper consultation’* to managing that same patient in a system with competing and ever-increasing demands.

### Theme 2: Time management

#### Personal time management

As discussed above, participants agreed that they need to work efficiently as there is often a limited time to complete tasks within a duty doctor session. Participants discussed the importance of making early decisions. For example, some participants discussed reviewing their list and selecting cases that were likely to require a face-to-face appointment or a home visit and dealing with these patients first. This allowed participants to plan when to review these patients within their session:

*‘I’ve seen registrars who they don’t get out till 7 o’clock on their afternoon on call because they’ve left phoning someone that I look at* [and] *thought that’s obviously a house call. I think why didn’t you phone that earlier on?’* (P1)

There was some debate among participants about entering the patient’s record to gain more information, to triage their workload. Some felt that, if they entered a patient record, they would deal with the scenario immediately, as this was the most time efficient way of managing their workload:

*‘Once you look in the notes, you’re going to phone them, because it takes time to call up a set of notes. So, once I’ve opened the notes, I’m dealing with that problem there and then.’* (P20)

Others valued the reassurance and reduction in cognitive load that entering a patient record might bring to their prioritisation decision making:

*‘… it makes me more anxious worrying about something that I haven't got to, that I'm worried I’m missing out on … just a bit of reassurance for myself.’* (P10)

Participants agreed that efficiency in personal time management is important, but how efficiency manifests might depend on the system and context in which the GP is operating. For example, if a GP is the duty doctor responsible for the practice’s acute presentations and carrying out home visits, which can be time consuming, they will organise their time differently from those who do not need to do home visits. Efficiency may also be more important to GPs who have responsibilities, for example, for childcare directly after their work shift. GPs who do not have these concerns could remain at work later without personal consequences:

*‘I see some of my partners who I know they’re juggling things, they’ve got child pick-up, and they get so uptight when they’re on call because they’re trying to get through everything.’* (P1)

#### System time management

Participants were aware that they were working in, and interacting with, a complex system in which the availability of resources varies throughout the day.

This included prioritising patients who required referral to another service to optimise their management, for example, to the ‘admission prevention team’ or ‘hospital at home’. For some, the earlier the referral was made, the more likely the referral was going to be accepted; for others, it provided an opportunity to gather further information should it be required. Other participants described prioritising those patients who might require medication:

*‘Because if you did have that mid-morning, you might be … more likely to phone and try and get some more information, because you might then be able to send the admission prevention team.’* (P9)

*‘But equally I wouldn’t want to leave stuff, people that might need prescriptions until right at the end, because then they might struggle to get them.’* (P7)

The nearest pharmacy might close before the GP practice, restricting the access to medication. The theme of time management is complex, with the participants not only considering their own time management or clinical urgency, but also what subsequently needs to happen for the desired outcome, which may be a patient receiving medication or secondary care accepting a referral.

### Theme 3: Experience

#### Familiarity with their patients’ histories

Participants rely on their familiarity with patients as a factor facilitating more time-efficient decision making, as GPs are already familiar with patients’ past medical history and social circumstances. Participants discussed being able to make quick decisions about patients’ presentations without needing to consult the medical record:

*‘I look at the list, and using the patient’s name, and my knowledge of the patient, and the problem, I will decide if they get a call back that day, or if I push them, put them somewhere else, if it’s more appropriate for them to be dealt with by someone else, or on another day.’* (P20)

Participants felt that this familiarity enabled them to manage patients without seeing them face to face. However, participants acknowledged that this took time to acquire and has come with spending time interacting with the same group of patients:

*‘And it’s hard to get that in twelve months, isn't it? Because if you’ve been in the practice for a decade, and you’ve met that patient over and over, it is difficult for* [registrars]*.’* (P30)

This is an important aspect when considering triage decision making. It would apply not only to GP registrars, but also locum doctors working with practices as well as recent recruits.

### Clinical experience and bias

Having worked for a prolonged period in general practice, participants acknowledged that they rely on their clinical experience from past presentations to make decisions. This bank of clinical experience influences how they make triage decisions. For example, one participant reflected that, when a patient presentation was not entirely in keeping with what they expected, they would manage that presentation differently from how they might ordinarily do so. This is likely pattern recognition that comes with experience:

*‘It’s experience, I suppose, yeah. That doesn’t sound right, let’s get them down.’* (P35)

The participants also acknowledged that their past experiences bring an element of bias, particularly when considering adverse events that may be more memorable:

*‘… mine is a bit of personal bias, having had an incident where it was a cardiac chest pain, so chest pain, I want to phone, just in case.’* (P9)

Consequently, GPs who have had significant events may subsequently triage patients differently from those who have not.

### Theme 4: Relationships

#### Trusting patient relationships

The participants reflected that experienced GPs have had time to develop therapeutic relationships with their patients:

*‘… if you know somebody who’s got COPD* [chronic obstructive pulmonary disease] *and they’re in hospital fairly regularly, if you know them well enough you can say well, do you feel you’re as bad as you were the last time you were in hospital? And you know how truthful they’re going to be.’* (P28)

Participants discussed that such relationships often result in a deeper understanding of the patient’s presentation, as highlighted in the quote above, and can enable more personalised decision making. These therapeutic GP–patient relationships can facilitate a certain level of trust; participants discussed relying on the histories that these patients have given them over the phone, rather than feeling that they must see them face to face.

#### Relationships with other team members

GPs work in small, interprofessional teams with considerable interdependence. Participants articulated the impact of requests made by other team members when making prioritisation decisions:

*‘For me, it’s about professional courtesy. If my district nurse is asking me urgently for a call ... that would be quite a high priority on my list.’* (P20)

Participants discussed prioritising requests made by other members of their team. They acknowledged the time pressure that colleagues may be under and discussed the importance of fostering good working relationships within their teams. Closely linked to this construct is the perception of competence of their colleagues:

*‘I know the nurses really well. So, I know which nurses I can have that sort of … “what do you think I should do” question to? And whatever they say I’m probably going to do.’* (P26)

The participants agree that, with good working relationships, they develop a deeper understanding of their colleagues’ competence and expertise, and consequently develop trust in their clinical judgement.

### Theme 5: Personal preferences

#### Stress and cognitive load management

##### Quick wins

There was some debate among participants about how they might prioritise less urgent tasks, particularly when it came to managing stress and cognitive load. Many discussed their desire to make their task list look more manageable by carrying out simpler and less time-consuming presentations first.

*‘Sometimes I go for a few quick wins if I’m feeling a little bit stressed. I think I know that person, I know what that’s about, I’ll just get that out the way and I’ll do that just to make it feel a bit more manageable.’* (P6)

##### Eating the frog

Others preferred doing the more difficult and least desirable tasks first as this lessened their cognitive load and enabled them to concentrate on the less taxing tasks thereafter:

*‘And that sometimes that comes into the sort of eating frogs, you know, when you get a list of jobs, one of them is eating frog, do that first, eat the frog first.’* (P16)

Acknowledging that approaches to stress and cognitive load management vary dependent on individual preferences and personalities is an important observation and one that must be considered when teaching triage decision making.

##### Self-care

Many participants recognised that repeatedly making decisions about clinical presentations resulted in a considerable cognitive burden; consequently, they had to ensure that they remained physically and mentally adept:

*‘So, I actually take a snack to eat at that time of the day, so I can get through to six. I find it affects my decision making if I’m too hungry.’* (P35)

##### Relationship with the SEIPS framework

A summary of the above themes and how they link to the SEIPS framework is detailed in Box 2.

**Box 2.** Summarising results of this study and mapping these to the SEIPS 2.0 framework themes

	Study theme	SEIPS themes	Summary
1	System awareness and adapting to demand	Organisation, person	GPs have an understanding of the constraints of the work system such as staffing levels and appointment availability, and change their working style to reflect this
2	Time management — personal time management — system time management	Task, organisation, tools, and technology	GPs are aware of the time constraints in their workplace and home life. This includes unpredictable demand, fixed surgery times, operating hours of other healthcare services, and IT system issues, and adjust their prioritisation decisions to reflect these
3	Experience — familiarity with patient histories — clinical experience — bias	Person	GPs’ longstanding knowledge of patient medical histories and consulting behaviours allows for quick risk assessment and decision making but can lead to some bias in decision-making processes
4	Relationships with staff and patients	Person and organisation	GPs rely on teamwork and communication among their own GP teams and trusting relationships among staff and also with patients to aid in their decision making
5	Personal preferences	Person	Individual GPs will have their own personal risk tolerances, consultation, and prioritisation styles

SEIPS = Systems Engineering Initiative for Patient Safety.

## Discussion

### Summary

This focus-group-based study explored the factors that experienced GPs consider when prioritising their workload. The following five key themes were identified: 1) system awareness and adapting to demand; 2) time management; 3) familiarity with patients; 4) relationships; and 5) personal preferences.

### Strengths and limitations

This is the first study to specifically explore the factors that GPs consider when making prioritisation decisions. It has contributed new information that may be of use to medical educators and those interested in designing GP triage systems.

The use of a TDG to form the basis of the focus groups is also a novel concept and one that may be of interest to future researchers exploring decision making and work systems.

The use of focus groups enabled the researchers to obtain a wide range of views. That said, the participants of the individual focus groups knowing each other could have had a positive or negative impact on participants’ ability to express their views, particularly around negative experiences.

The use of the SEIPS framework is also a novel way to explore decision making as it situates it within the work system. This allowed us to explore why decisions are made in the way that they are and provides possible areas of intervention to improving decision-making processes or to develop future teaching tools.

Although the participants came from diverse practice groups, they all worked in central Scotland, which may impact on the transferability of the research findings.

The influence of comorbidity and different cultural groups on prioritisation was not covered in the focus groups and would have added to the research. An observational component to the study would also have added a greater richness to the data obtained.

### Comparison with existing literature

It is of interest to consider the tensions that exist within these themes in making decisions regarding patient care, for example, the tension between meeting the needs of the individual patient versus the entire patient population and balancing the needs of patients versus the needs of the team members (Figure 1). GPs must carefully balance these tensions when making prioritisation decisions.

The consultation remains at the heart of a GP’s work and is therefore the key area to be altered depending on demand. In this study, GPs noted that a flexible approach to their consultation style was one of the ways that they coped with increasing patient complexity and demand. However, GP consultation skills are often taught in a prescriptive way with the consultation requiring multiple elements for it to be deemed an optimal interaction, with little room for variation.^13–15^ This encourages rigidity and highlights a gap between the skills obtained through training and the skills required to operate in real-world scenarios.^16^ These models often give a sense of there being a right and wrong way to consult, which may stifle the ability to adapt to demand.

Evidence suggests that time allocated to consulting is often not adequate to meet the demands placed on it by national guidelines, yet training does not take this into account.^17,18^ GPs who are under time pressure tend to adopt a more doctor-centred, biomedical, and transactional consultation style and leave less time to address psychological issues within their consultations.^19^ Studies have suggested a decrease in patient and doctor satisfaction with this approach to consulting.^20^ Yet, to meet the needs of the practice population these tensions must be overcome so that health care is provided to those that most require it. It is important that GP registrars are trained to recognise when a work system is under significant pressure and be aware of the potential need to adapt their consultation style to meet this demand while practising safely.

Another finding from this study was the differences in GPs’ cognitive load management. Cognitive overload is a state in which information retention, processing, and task performance is impeded owing to a saturation of the individual’s working memory.^21^ There is an association between cognitive overload and poor performance in work-related tasks.^22,23^ GPs are often presented with a large list of tasks that will have varying degrees of perceived urgency and may seem daunting.

In this study there were differing strategies in task management among participants. Some participants went for the ‘quick-wins’ approach. This means they sought out and completed the easier tasks that would require less cognitive effort and did these first, meaning that the list felt more manageable. This approach has the benefit of generating momentum in the completion of the tasks and makes the overall size of the task seem more manageable. Evidence from cognitive neuroscience suggests that the dopamine release from the completion of smaller tasks can help reinforce engagement and improve mental energy levels.^24^

Other participants tended to adopt an ‘eat-the-frog’ approach to the management of their task list. Using this strategy, participants sought out and completed the most difficult task first. Difficult tasks might involve possible confrontation or where the task was perceived to take a lot of time, and therefore the ones that clinicians may be more likely to procrastinate over. The phrase ‘eating the frog’ is attributed to a quote from the author Mark Twain: *‘Eat a live frog first thing in the morning, and nothing worse will happen to you the rest of the day*.*’* Proponents of this approach point out that carrying out the most demanding task first will mean the effort that the task requires is more likely be given to it. Starting a session in an impactful way can also boost morale.^25^ It is important that GPs are aware of their own preferences in managing their cognitive load. It may be helpful for GP registrars and educational supervisors to discuss registrars’ preferences to help them to understand the advantages and disadvantages of each approach to promote adaptability in prioritisation.

### Implications for research and practice

Future research could explore the impact of prioritisation decisions on patient outcome, for example, does one type of decision making result in an increase in hospital admissions? Work could also explore the factors that other team members, particularly in the administration team, consider when making appointment allocation decisions. The influence of personality type on prioritisation strategies could also be an interesting area of research. Furthermore, the use of TDGs in the teaching of prioritisation to GP registrars, experienced GPs, and wider primary care team members is also an area of potential future research interest, with TDG responses by experienced GPs informing the teaching content.

In conclusion, prioritisation is a complex and increasingly important skill in modern general practice. It is important that GP registrars and other staff involved in prioritisation are given the time to develop the skills required to prioritise effectively and be aware of the pros and cons of implementing various triaging strategies. It is also important that there is a recognition of the dilemmas that GPs face in prioritisation and that strategies are developed and taught in GP training programmes to confront these. We hope that the findings of this study will enable educators to design resources to help GPs, GP registrars, and other primary care staff to develop these skills.
